# Impact of Ring Finger Protein 20 and Its Downstream Regulation on Renal Tubular Injury in a Unilateral Nephrectomy Mouse Model Fed a High-Fat Diet

**DOI:** 10.3390/nu15234959

**Published:** 2023-11-29

**Authors:** You-Jin Kim, Se-Hyun Oh, Jeong-Hoon Lim, Jang-Hee Cho, Hee-Yeon Jung, Chan-Duck Kim, Sun-Hee Park, Tae-Hwan Kwon, Yong-Lim Kim

**Affiliations:** 1 Department of Internal Medicine, School of Medicine, Kyungpook National University, Daegu 41944, Republic of Korea; youjin@knu.ac.kr (Y.-J.K.); sehyun.oh@knu.ac.kr (S.-H.O.); jh-cho@knu.ac.kr (J.-H.C.); hy-jung@knu.ac.kr (H.-Y.J.); drcdkim@knu.ac.kr (C.-D.K.); sh-park@knu.ac.kr (S.-H.P.); 2 Cell and Matrix Research Institute, Kyungpook National University, Daegu 41944, Republic of Korea; 3 Division of Nephrology, Department of Intermanl Medicine, Kyungpook National University Chilgok Hospital, Daegu 41404, Republic of Korea; 4 Department of Biochemistry and Cell Biology, School of Medicine, Kyungpook National University, Daegu 41944, Republic of Korea; thkwon@knu.ac.kr

**Keywords:** ring finger protein 20, lipid metabolism, kidney damage, peroxisome proliferator-activated receptor signaling

## Abstract

Abnormal lipid metabolism increases the relative risk of kidney disease in patients with a single kidney. Using transcriptome analysis, we investigated whether a high-fat diet leads to abnormalities in lipid metabolism and induces kidney cell-specific damage in unilateral nephrectomy mice. Mice with unilateral nephrectomy fed a high-fat diet for 12 weeks exhibited progressive renal dysfunction in proximal tubules, including lipid accumulation, vacuolization, and cell damage. Ring finger protein 20 (RNF20) is a ligase of nuclear receptor corepressor of peroxisome proliferator-activated receptors (PPARs). The transcriptome analysis revealed the involvement of RNF20-related transcriptome changes in PPAR signaling, lipid metabolism, and water transmembrane transporter under a high-fat diet and unilateral nephrectomy. In vitro treatment of proximal tubular cells with palmitic acid induced lipotoxicity by altering RNF20, PPARα, and ATP-binding cassette subfamily A member 1 (ABCA1) expression. PPARγ and aquaporin 2 (AQP2) expression decreased in collecting duct cells, regulating genetic changes in the water reabsorption process. In conclusion, a high-fat diet induces lipid accumulation under unilateral nephrectomy via altering RNF20-mediated regulation and causing functional damage to cells as a result of abnormal lipid metabolism, thereby leading to structural and functional kidney deterioration.

## 1. Introduction

The association between kidney disease and lipid metabolism abnormalities complicates the prognosis of chronic kidney disease (CKD) [1,2]. The mechanism underlying renal function deterioration in obese patients with CKD remains unclear despite obesity being a well-documented risk factor for kidney damage [3,4]. The kidney stands as a complex organ with distinct cell distribution and functions, and damage can result from various causes [5]. The proximal tubule (PT), comprising the bulk of renal tissue, accounts for approximately 60% of the kidney’s energy metabolism [6,7,8]. Meanwhile, collecting duct (CD) cells specialize in water reabsorption and contribute significantly to maintaining osmotic pressure and overall homeostasis [9]. Given these factors, it is expected that lipid accumulation within renal tissues due to a high-fat diet and abnormal lipid metabolism may have varying impacts on different cell types, potentially leading to the deterioration of kidney function.

The ring finger protein 20 (RNF20), a transcriptional regulator and E3-ubiquitin ligase known for its multifaceted roles in DNA damage response [10], stem cell differentiation [11], and lipid biosynthesis [12], was investigated in this study. RNF20-induced SREBP1c ubiquitination downregulates hepatic lipogenic activity and reduces renal cancer metastasis [13]. RNF20 promotes peroxisome proliferator-activated receptor gamma (PPARγ) activity to reduce adipocyte size [14]. This suggests that RNF20 plays an important role in regulating lipid metabolism and renal function, including PPAR regulation. However, no study has reported on the correlation between lipid metabolism abnormalities and kidney cell damage in relation to RNF20. The abundant presence of PPARγ, which is essential for lipid metabolism and sodium and water transport through aquaporin-2 (AQP2) proteins, within CD cells is particularly interesting [15,16]. A recent study revealed the role of AQP2 in cholesterol-related natriuretic dysfunction, further underscoring the interconnectedness of these processes [17]. These observations led to the hypothesis that lipid accumulation within PT and CD cells may catalyze lipid-mediated energy metabolism and AQP2-mediated sodium and water transport abnormalities through RNF20-mediated regulation in PPARs, ultimately contributing to the decline in renal function.

The present study aimed to determine the mechanisms underlying lipid metabolism abnormalities and natriuretic disorders in renal damage caused by obesity in patients with CKD through RNF20 regulation.

## 2. Materials and Methods

### 2.1. Animals

Five-week-old male C57BL/6 mice (Hyochang Science, Dague, Republic of Korea) were randomly divided into two groups based on dietary intake: a normal diet group (ND; D10001, Research Diets, New Brunswick, NJ, USA) and a high-fat diet group (HD, D12451, Rodent Diet with 45 Kcal % Fat, Research Diets, New Brunswick, NJ, USA). These diets were maintained throughout 14 weeks. After 2 weeks of diet, randomized mice were subjected to uninephrectomy (UN) on week 3. The groups were labeled as follows: ND, normal diet (*n* = 5); NDU, normal diet + uninephrectomy (*n* = 5); HD, high-fat diet (*n* = 8); and HDU, high-fat diet + uninephrectomy (*n* = 8). After anesthetizing the mice with 3–5% isoflurane, the left kidney was surgically removed. Thirteen weeks after surgery, all mice were sacrificed, and kidneys were collected for analysis. Half of the kidneys were used for molecular analysis, and the other half were used for histological analyses. Animal experiments were performed according to the guidelines approved by the Animal Care and Use Committee at the Kyungpook National University (KNU-2021-0179, KNU-2023-0133).

### 2.2. Biochemical and Histopathological Studies

Blood urea nitrogen (BUN), creatinine (Cr), cholesterol, LDL cholesterol, HDL cholesterol, triglyceride (TG), glucose (Glu), albumin (Alb), protein (Pro), alanine aminotransferase (ALT), and aspartate aminotransferase (AST) levels in mouse serum were evaluated by GCLabs (Yongin, Republic of Korea) using the Cobas 8000 modular analyzer system (Roche, BW, Germany). Kidney tissues from each experimental group were immersion-fixed with 4% paraformaldehyde (pH 7.4) and embedded in paraffin. Four-micrometer tissue sections were prepared and stained with periodic acid–Schiff (PAS) stain using standard protocols to determine histological characteristics. Immunohistochemical analysis of the kidney tissues detected the RNF20 protein (1:100, PA5-34552, Thermo Fisher, Waltham, MA, USA). Further staining was performed using osmium tetroxide to better distinguish LDs. Small pieces (3 mm^2^) of kidney tissue were first fixed in 2.5% glutaraldehyde (Sigma-Aldrich, St. Louis, MO, USA) for 2 h and then in 1% osmium tetroxide (Merck, Darmstadt, Germany) for 90 min. Staining with osmium tetroxide revealed the accumulation of lipid droplets in the kidney of mice from each group.

### 2.3. RNA Sequencing and Analysis

Mice (operation after 12 weeks; male; ND, NDU, HD, and HDU groups) were deeply anesthetized with isoflurane and sacrificed after cardiac transfusion. Their kidney tissues were immediately placed in RNAlater solution (Ambion) and stored overnight at 4 °C. One kidney per mouse was used to generate one RNA sample, and three sets of RNA samples (three mice per group) were processed for RNA sequencing. RNA extraction, library preparation, cluster generation, and sequencing were performed by Macrogen Inc. (Seoul, Republic of Korea). RNA samples for sequencing were prepared using the TruSeq Stranded mRNA Library Prep Kit (Illumina platform) according to the manufacturer’s instructions.

Original raw data were targeted at read count values for known genes obtained through the StringTie “-e” option. Paired-end transcriptome sequencing of 12 samples revealed that they were within the normal range. After filtering out low-quality genes during data preprocessing and quality check, relative log expression normalization was performed. The raw data for each sample and the trimmed reads that went through the preprocessing process were compared to the total data amount and Q30 (phred score, base quality ≥ 30) values. Trimmed reads that were processed for spliced read mapping through the Bowtie2 aligner were mapped to the known *Mus musculus* genome (mm10) using the HISAT2 program. After read mapping, transcript assembly was performed using the StringTie program. Consequently, expression profile values were obtained for each sample for known transcripts, and read count, FPKM (fragment per kilobase of transcript per million mapped reads), and transcripts per kilobase million values were organized based on transcript/gene. Statistical analysis used fold change and the nbinomWaldTest in DESeq2 for each comparative combination. Using this value, differentially expressed gene (DEG) analysis was performed using DESeq2 for four comparison combinations (ND, NDU, HD, and HDU), and |fc| ≥ 2 and nbinomWaldTest raw in at least one comparison combination. We extracted 2577 genes that had a *p* value of <0.05. Heatmaps helped visualize expression data grouped by sample and gene using the degree of similarity of each gene’s expression pattern in each sample through hierarchical clustering (distance metric: Euclidean distance; linkage method: complete) of significant genes. Functional annotation was performed using Gene Ontology (GO) and Kyoto Encyclopedia of Genes and Genomes (KEGG). For significant DEGs, gProfiler (https://biit.cs.ut.ee/gprofiler/orth, accessed on 25 September 2022) was used for the functional classification of GO results into biological processes (BPs), molecular functions, and cellular components (CCs). We also conducted individual gene set enrichment analyses. All analyses were performed by Macrogen Inc. (Seoul, Republic of Korea).

### 2.4. Cell Culture Treatment

HK2 (CRL-2190™) and M1 (CRL-2038™) cells were purchased from the American Type Culture Collection (Manassas, VA, USA). HK2 cells were cultured in a medium containing RPMI-1640, 10% fetal bovine serum (FBS), 100 U/mL penicillin, and 100 µg/mL streptomycin at 37 °C in 5% CO_2_. M1 cells were cultured in a medium containing a 1:1 mixture of Dulbecco’s modified Eagle’s medium and Ham’s F12 medium, 5% FBS, 100 U/mL penicillin, and 100 µg/mL streptomycin at 37 °C in 5% CO_2_. The cultured HK2 and M1 cells were starved under serum-deprived conditions for 24 h and then treated with palmitic acid (PA) (P9767, Sigma-Aldrich, St. Louis, MO, USA) for 24 h. Then, conjugated with 10% fatty acid-free bovine serum albumin (BSA) (A7030, Sigma-Aldrich, St. Louis, MO, USA) and 40 mM PA at a 4:1 ratio, a final concentration BSA-conjugated PA of 8 mM was diluted in RPMI-1640 or DMEM/F12 medium for further experiments.

### 2.5. Lipid Accumulation

HK2 and M1 cells were seeded in an 8-well chamber and treated with PA for 24 h. Cells were rinsed in PBS and incubated in 4% paraformaldehyde for 30 min at 4 °C. Cells were then washed with ddH_2_O twice and 60% isopropanol for 5 min at room temperature. After the cells dried completely, they were stained with the Oil-Red O working solution for 30 min at room temperature. The Oil-Red O solution was removed, and the cells were immediately washed four times with ddH_2_O. Nuclear staining was performed with hematoxylin.

### 2.6. Western Blot

For immunoblot analysis, 10 µg of protein was separated via 10% SDS-polyacrylamide gel electrophoresis and transferred to a nitrocellulose membrane. The membrane was then blocked with 10% skimmed milk for 1 h at room temperature and incubated overnight at 4 °C with primary antibodies. The next day, the samples were incubated with a horseradish peroxidase-conjugated secondary antibody (Dako, Glostrup, Denmark) for 1 h at room temperature, and the signal was detected using advanced ECL reagents (Cytiva, Marlborough, MA, USA). The signal intensity was quantified using the Scion Image software (Scion Corp., Frederick, MD, USA). The primary antibodies used are listed in Table 1.

### 2.7. Statistical Analysis

Data are represented as mean ± standard error of the mean (SEM). Statistical analyses were performed using GraphPad Prism 8 (GraphPad Software, San Diego, CA, USA). Differences among the experimental groups were analyzed using one-way nonparametric ANOVA, followed by Tukey’s multiple comparison test. Multiple comparison tests were only performed when a significant difference was determined using ANOVA (*p* < 0.05).

## 3. Results

### 3.1. Changes in Lipids and Kidney Function Caused by a High-Fat Diet and Uninephrectomy

**Figure 1:**
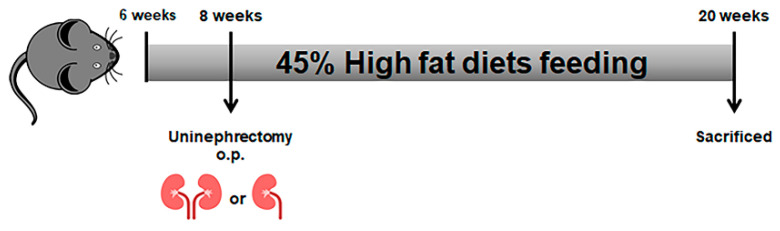
Animal experiment timeline.

After unilateral nephrectomy to induce kidney damage, normal and high-fat diets were administered for 12 weeks to determine changes in lipid composition and kidney function according to the diet type in mice (Figure 1). Comparison between the bilateral and unilateral kidney models confirmed a reduction in body weight in the NDU group compared with the ND group. However, no differences were observed between the HD and HDU groups (Table 2). Blood biochemistry test results confirmed that BUN and Cr were significantly increased in mice that underwent unilateral nephrectomy compared with those with bilateral kidney. In particular, the HDU group measured significantly higher than the NDU and HD groups. Cholesterol and LDL levels were significantly increased in the HD and HDU groups compared with the ND and NDU groups. The levels of Glu, Alb, Pro, and liver function were not significantly different among the groups (Table 3).

### 3.2. Genome-Wide Transcriptome Analysis Associated with the Acceleration of Kidney Injury Progression by High-Fat Diet and Uninephrectomy

**Figure 2:**
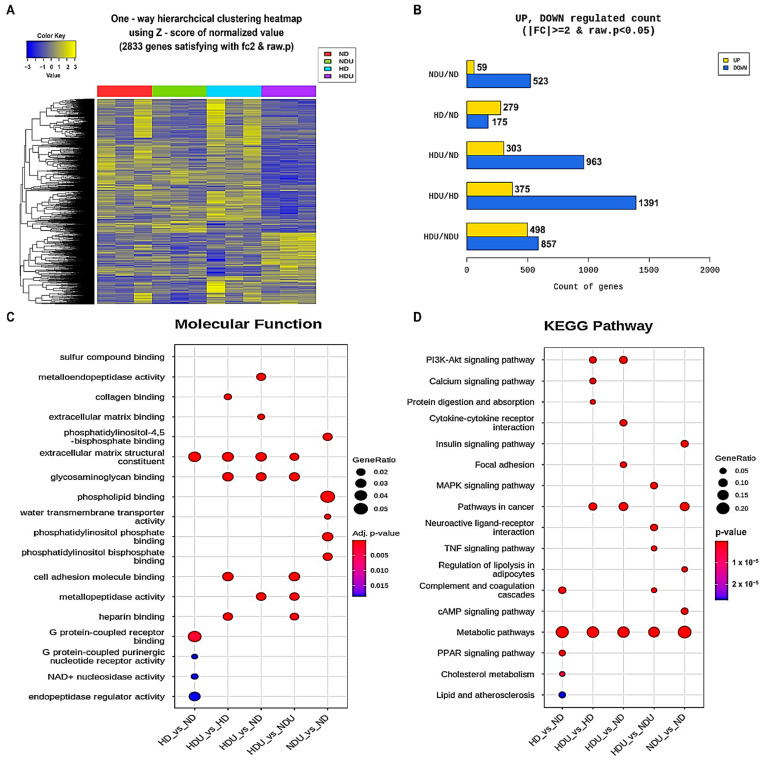
Results of GO and KEGG analyses. (**A**) Clustering heatmap after DEGs between groups (**B**) Number of upregulated and downregulated DEGs. (**C**) GO analysis to identify the molecular function genes associated with high-fat-induced kidney injury in the unilateral kidney. (**D**) KEGG analysis to identify DEGs between groups. *n* = 3 for each group.

We conducted an analysis of transcriptional changes in kidney tissue in response to uninephrectomy and high-fat diet. DEG analysis data were presented using cluster analysis and confirmed changes in bilateral or unilateral kidney function and gene expression depending on the diet. In the HDU group, the gene expression pattern revealed a distinct downregulation of numerous genes compared with the other groups (Figure 2A). Interestingly, a higher number of genes was downregulated in unilateral kidney groups (NDU and HDU). The most significant changes were observed between the HDU and HD groups, with 375 being upregulated and 1391 being downregulated, out of 1766 genes. Comparative analysis of the ND and HD groups revealed that 279 genes were upregulated and 175 downregulated in the HD group compared with the ND group (Figure 2B).

Next, GO analysis was performed to determine the molecular function in the kidney related to the interaction between unilateral nephrectomy and diet. This analysis revealed that genes involved in kidney development, renal system regulation, cell adhesion, and cellular structure formation and maintenance (extracellular matrix binding and structural constituent) were relevant in all groups. In comparison with the ND group, the HD group exhibited a clear correlation with genes regulating G protein binding and enzyme/regulator activity (G protein receptor binding and activity, NAD+ nucleosidase activity, and endopeptidase regulator activity). Compared with the HD group, the HDU group was associated with genes related to cell maintenance functions (cell adhesion molecule and collagen and glycosaminoglycan binding). Interestingly, compared with the ND group, genes affecting water transmembrane transporter activity and phospholipid binding were detected in the NDU group (Figure 2C).

In the KEGG pathway analysis based on GO analysis results, genes involved in the metabolic pathway were identified in all groups. Compared with the ND group, the HD group was associated with PPAR signaling, cholesterol metabolism, lipid and atherosclerosis, and metabolic pathways. In particular, the comparative analysis of the HDU and NDU groups revealed involvement in MAPK signaling, TNF signaling, and complement and coagulation cascade pathways. The comparative analysis of the NDU and ND groups confirmed that insulin signaling, regulation of lipolysis in adipocytes, and cAMP signaling were related, whereas the comparative analysis of the HDU and HD groups revealed that PI3K-Akt signaling, calcium signaling, and protein digestion and absorption were related. Our analysis revealed changes in lipid metabolism-related genes induced by a high-fat diet, and concomitantly, the induction of unilateral nephrectomy confirmed associations with cell survival and apoptosis, cell cycle, and inflammatory factor pathways. We focused on RNF20, which is associated with these pathways and has the potential to induce protein regulation, and associations with PPARs in the HD group and water transmembrane transporters in the NDU group (Figure 2D). Related gene changes were confirmed based on the KEGG analysis (Table 4).

### 3.3. Changes in Protein Expression by Kidney Cell Types Due to High-Fat Diet and Unilateral Kidney Injury

**Figure 3:**
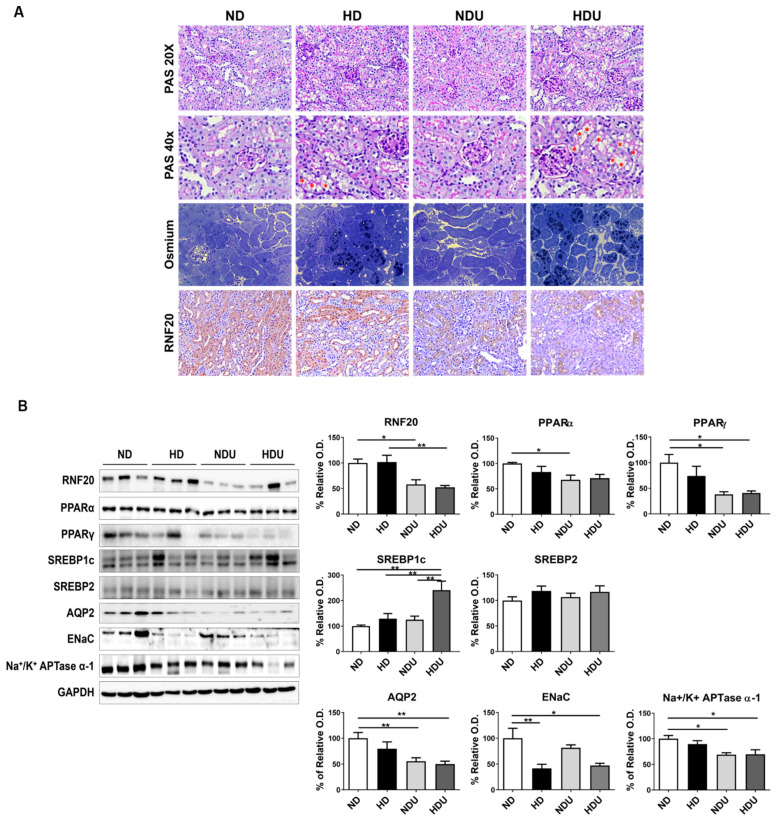
Changes in protein expression by kidney cell types due to unilateral kidney injury and high-fat diet. (**A**) Representative histological photomicrograph of periodic acid–Schiff (PAS), osmium (40×), and RNF20 (20×) staining in kidney tissue. * Indicates vacuoles in PAS staining. Black indicates lipid droplets in osmium staining. (**B**) Representative Western blot and densitometric analysis of RNF20, PPARα, PPARγ, SREBP1c, SREBP2, AQP2, ENaC, and Na^+^/K^+^ ATPase α-1 protein levels in kidney tissues. Relative protein expression was determined using densitometry. The difference among the groups was analyzed using one-way nonparametric ANOVA followed by Tukey’s multiple comparison test. Each bar represents mean ± SEM. * *p* < 0.05, ** *p* < 0.01 versus ND. *n* = 3–5 for each group.

The mechanisms underlying renal function decline and exacerbation as a result of high-fat diet appear to vary in tubular cells. Thus, in the present study, we confirmed changes in PT and CD cells, which constitute the kidney and exhibit different characteristics. Regardless of the diet, PAS staining confirmed PT damage in uninephrectomy groups (NDU and HDU) compared with sham groups (ND and HD). The high-fat diet was observed to induce vacuole formation in the tubular cells of the HD and HDU groups. Examination of lipid droplets in cells using osmium staining confirmed increased lipid accumulation and vacuole formation in the renal tubules of the HDU group. RNF20 expression was confirmed in PT, and no differences were observed between the HD and ND groups; however, RNF20 expression was decreased in the NDU and HDU groups (Figure 3A).

As a result of identifying the PPAR family related to the expression of RNF20 and AQP2, PPARα tended to decrease in all groups compared with the ND group and significantly decreased in the NDU group. PPARγ expression was significantly decreased in the NDU and HDU groups compared with the ND group. Regarding SREBP protein changes, the expression of SREBP1c was significantly increased in the HDU group compared with all groups, but no differences were observed in SREBP2 expression. AQP2 expression was decreased in the HDU group compared with the ND group, and ENaC expression was significantly decreased in the HD and HDU groups. Na^+^/K^+^ APTase α-1 level was also decreased in the NDU and HDU groups compared with the ND group (Figure 3B).

### 3.4. Lipid Accumulation Due to RNF20-Related Lipid Metabolism Changes in PT Cells

**Figure 4:**
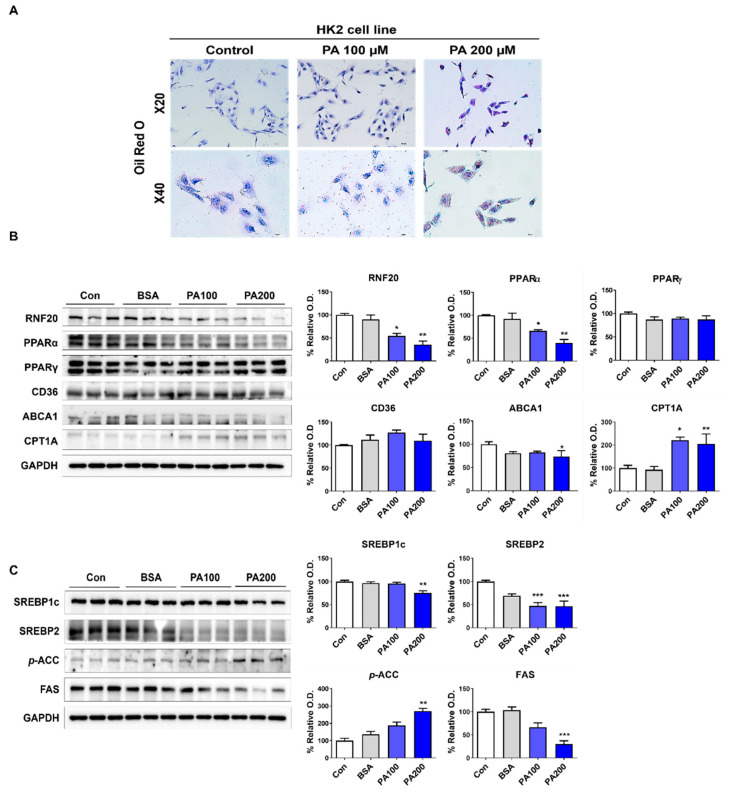
Lipid accumulation due to RNF20-related lipid metabolism changes in PT cells. (**A**) Representative images of Oil-red O staining of HK2 cells. (**B**) Representative Western blot and densitometric analysis of RNF20, PPARα, PPARγ, CD36, ABCA1, and CPT1A protein levels in HK2 cells with/without PA. (**C**) Representative Western blot and densitometric analysis of SREBP1c, SREBP2, *p*-ACC, and FAS protein levels in HK2 cells with/without PA. The difference among the groups was analyzed using one-way nonparametric ANOVA followed by Tukey’s multiple comparison test. Each bar represents mean ± SEM. * *p* < 0.05, ** *p* < 0.01, *** *p* < 0.001 versus control. *n* = 3–5 for each group.

The transcriptomic and protein analyses of the whole kidney suggested that high-fat diet promotes kidney damage through different damage mechanisms in PT and CD. These results confirmed that changes in nuclear transcription factors associated with a high-fat diet and kidney-regulated metabolism due to unilateral nephrectomy affect different cell types. First, PA-induced lipid accumulation was confirmed in HK2 cells, the PT responsible for the kidney’s energy source (Figure 4A). Next, the expression of related proteins, RNF20, PPARα, and ABCA1, was significantly reduced, whereas the expression of PPARγ and CD36 was not different from that observed in the control group. The expression of CPT1A and *p*-ACC was significantly increased following treatment with 200 µM PA in HK2 cells, whereas that of SREBP1c, SREBP2, and FAS was reduced (Figure 4B).

### 3.5. Lipid-Induced Changes in Nuclear Receptor Factors and Water Channel-Related Proteins in CD Cells

**Figure 5:**
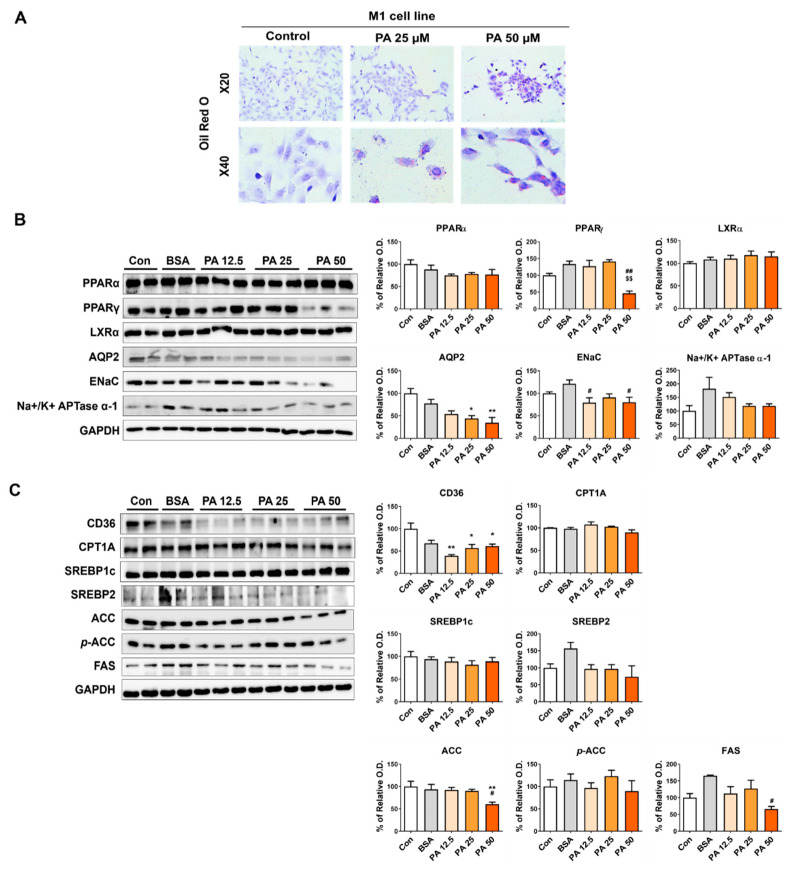
Lipid-induced changes in nuclear receptor factors and water channel-related proteins in CD cells. (**A**) Representative images of Oil-red O staining of M1 cells. (**B**) Representative Western blot and densitometric analysis of PPARα, PPARγ, LXRα, AQP2, ENaC, and Na^+^/K^+^ ATPase α-1 protein levels in M1 cells with/without PA. (**C**) Representative Western blot and densitometric analysis of CD36, CPT1A, SREBP1c, SREBP2, ACC, *p*-ACC, and FAS protein levels in M1 cells with/without PA. The difference among the groups was analyzed using one-way nonparametric ANOVA followed by Tukey’s multiple comparison test. Each bar represents mean ± SEM. * *p* < 0.05, ** *p* < 0.01 versus control; ^#^ *p* < 0.05, ^##^ *p* < 0.01 versus BSA; ^$$^ *p* < 0.01 versus PA 25 μM. *n* = 3–5 for each group.

When M1 cells, which are CD cells essential for homeostasis maintenance, were stimulated with PA, changes in lipid metabolism and water channel proteins were observed. PA induced lipid accumulation in M1 cells (Figure 5A). The expression of related proteins, PPARα and LXRα, did not differ from that in the control group. PPARγ expression significantly decreased at 50 µM of PA, and the related AQP2 expression significantly decreased. ENaC expression was significantly reduced in the PA-treated group, and Na^+^/K^+^ APTase α-1 displayed no difference between the PA-treated and control groups (Figure 5B). CD36 expression decreased in the PA-treated group compared with the control group. FAS and ACC exhibited a significant decrease in expression only at 50 µM of PA; however, the expression of CPT1A, SREBP1c, SREBP2, and *p*-ACC did not differ from that in the control group (Figure 5C).

## 4. Discussion

The relationship between kidney disease and lipid metabolism abnormalities increases the relative risk of the occurrence of worsening kidney diseases depending on blood cholesterol levels, including LDL, HDL, and TG levels [18,19]. Clinically, an association between lipid accumulation and kidney damage after nephrectomy has been reported, but the exact mechanism remains unclear. The presented results emphasize the intricate interplay among diet, kidney function, gene expression, and protein expression under high-fat diet and unilateral nephrectomy conditions. These findings have significant implications for understanding the mechanisms underlying kidney damage and progression to dysfunction under these conditions.

Examination of changes in lipid levels and kidney function revealed a reduction in body weight in the NDU group compared with the ND group, which can be attributed to the altered kidney function following unilateral nephrectomy. The increase in BUN and Cr levels in the unilateral nephrectomy groups (NDU and HDU), particularly the HDU group, indicates impaired kidney function, potentially resulting from the cumulative effects of HD and nephrectomy. The significant increase in cholesterol and LDL levels in high-fat diet groups (HD and HDU) suggests the role of diet in lipid metabolism.

A transcriptome analysis was performed in four groups to determine changes in gene expression according to lipid accumulation in the kidney. The analysis revealed extensive changes in gene expression patterns influenced by diet and nephrectomy. The HDU group exhibited the most significant gene expression changes, with numerous genes downregulated. The analysis also identified various pathways and BPs affected by these changes. The involvement of pathways such as PPAR signaling, protein and cholesterol metabolism, and water transmembrane transport underscores the impact of high-fat diet on kidney health.

Using transcriptome analysis, lipid metabolism associated with a high-fat diet as well as renal functions, including PPAR signaling, lipid metabolism, and water homeostasis regulation, with a specific focus on RNF20, were investigated; moreover, the genes and proteins associated with the abovementioned processes as well as their changes were explored. RNF20 acts as an E3-ubiquitin ligase and regulates histone H2B monoubiquitination (H2Bub1) [20]. Through this modification, RNF20 reduces cell size by inactivating PPARγ in adipocytes [21] and suppresses metastasis by regulating SREBP1c in cancer cells [13,22]. These findings suggest that the regulation of RNF20 is associated with PPARs and lipid metabolism in various cells. However, research on the role of RNF20 in kidney cells is still limited.

The significant change in gene expression indicated that RNF20 expression was decreased in the high-fat diet and nephrectomy models (HD and HDU), and the PPARα and PPARγ expression was decreased in the nephrectomy models (NDU and HDU). Nuclear receptor corepressor (NcoR) regulates the activity of PPARα and PPARγ in adipocytes, immune cells, and the liver [23], and is degraded by the proteasome due to E3-linked ubiquitination by RNF20 [14]. The transcriptome analysis of kidney tissue indicated that *NcoR* was upregulated in the HDU group compared with the HD and NDU groups, demonstrating that lipid metabolism regulation through the nuclear transcription factor activity of *NcoR* occurs not only in adipocytes, but also in kidney cells. Moreover, PPARα activity inhibition and increased SREBP1 expression by RNF20 indicated that high-fat diet-induced lipid accumulation and cellular lipid imbalance occurred in mice who had undergone nephrectomy, suggesting that RNF20-dependent PPAR signaling is one of the several renal cell injury mechanisms.

The kidney differentially expresses all PPAR isoforms [24]. PPARα is highly expressed in the PT and promotes the expression of genes involved in fatty acid transport and oxidation [25]. PPARγ is expressed in the distal tubules and CD, and regulates glucose metabolism, sodium reabsorption, and inflammation in the renal system [26]. AQP2, a water channel protein expressed in the CD, is regulated by arginine vasopressin (AVP) and is essential for regulating water reabsorption [27]. Our analyses confirmed that AQP2 was downregulated in nephrectomy mice fed a high-fat diet. In addition, the expression of genes involved in the vasopressin-regulated water reabsorption pathway, which directly regulates AQP2, was downregulated due to the effects of a high-fat diet and nephrectomy. These results suggest that AQP2 reduction through vasopressin regulation and PPARγ may contribute to the worsening of renal damage during natriuretic dysfunction, a renal tubular deformation associated with abnormalities in lipid metabolism.

The different effects observed in PT and CD cells of the kidney suggest distinct mechanisms underlying renal function decline. Kidney tubule damage and loss were more pronounced in the HDU group, indicating a more severe impact of combining unilateral nephrectomy with a high-fat diet. Changes in the expression of RNF20, AQP2, PPARα, and PPARγ indicated that they contributed to maintaining and regulating renal function. The confirmation of lipid accumulation in HK2 cells, representing PT cells, further supports the notion that lipid metabolism plays a key role in kidney damage. The downregulation of RNF20, PPARα, and ABCA1 suggests that lipid metabolism pathways are perturbed, potentially contributing to lipid accumulation in PT cells. The findings related to M1 cells, which represent CD cells, highlight the intricate interactions between lipid metabolism and water channel proteins. The downregulation of PPARγ and AQP2 suggests an impaired water channel function, potentially impacting water balance, and decreases in ENaC expression may further contribute to kidney dysfunction.

The limitation of this study is that while we identified RNF20-related kidney cell damage, in vivo experiments using an RNF20 overexpression model were not performed.

## 5. Conclusions

Our results suggest that reduced expression of RNF20 due to a high-fat diet and unilateral nephrectomy has complex and distinct effects on various aspects of renal function by downregulating PPAR signaling, including lipid metabolism gene and protein expression, in different renal cell types. The expression of RNF20 in the kidney contributes to the decline in renal function and is believed to have significant implications for understanding renal diseases involving metabolic factors.

## Figures and Tables

**Table 1:** List of primary antibodies used in the study.

Primary Antibody	Host	Dilution	Cat. No.	Source
PPARγ (C26H12)	Rabbit	1:1000	2435s	Cell Signaling Technology, Danvers, MA, USA
LXRα	Rabbit	1:1000	ab176323	Abcam, Cambridge, UK
CD36	Rabbit	1:1000	ab133625	Abcam, Cambridge, UK
ABCA1	Mouse	1:1000	ab18180	Abcam, Cambridge, UK
PPARα	Mouse	1:500	SC-398394	Santa Cruz Biotechnology, Santa Cruz, CA, USA
CPT1A	Mouse	1:1000	ab128568	Abcam, Cambridge, UK
Fibronectin	Rabbit	1:1000	ab2413	Abcam, Cambridge, UK
RNF20	Rabbit	1:1000	Ab32629	Abcam, Cambridge, UK
AQP2	Mouse	1:1000	SC-515770	Santa Cruz Biotechnology, Santa Cruz, CA, USA
ENaC	Rabbit	1:1000	PA1-920A	Invitrogen, Grand Island, NY, USA
SREBP1	Mouse	1:500	SC-365513	Santa Cruz Biotechnology, Santa Cruz, CA, USA
SREBP2	Mouse	1:500	SC-13552	Santa Cruz Biotechnology, Santa Cruz, CA, USA
Acetyl-CoA Carboxylase (C83B10)	Rabbit	1:1000	3676S	Cell Signaling Technology, Danvers, MA, USA
Phospho-Acetyl-CoA Carboxylase (Ser79)	Rabbit	1:1000	3661s	Cell Signaling Technology, Danvers, MA, USA
Fatty Acid Synthase (C20G5)	Rabbit	1:1000	3180s	Cell Signaling Technology, Danvers, MA, USA
Na^+^/K^+^ATPase α1	Mouse	1:1000	05-369	Sigma-Aldrich, St. Louis, MO, USA
GAPDH	Rabbit	1:5000	2118	Cell Signaling Technology, Danvers, MA, USA

**Table 2:** Body weight measurement.

Weeks	ND	NDU	HD	HDU
Before 2 weeks	20.0 ± 0.2	20.2 ± 0.2	19.0 ± 0.4	20.3 ± 0.4
Uninephrectomy o.p. weeks	24.4 ± 0.8	23.4 ± 0.4	23.7 ± 1.1	25.5 ± 0.6
After 2 weeks	27.7 ± 0.9	23.7 ± 0.5	25.7 ± 0.9	26.0 ± 0.7
After 4 weeks	29.1 ± 0.8	25.4 ± 0.5	29.0 ± 1.2	28.5 ± 0.7
After 6 weeks	31.5 ± 0.8	28.1 ± 0.5	34.7 ± 2.0	32.4 ± 0.7
After 8 weeks	33.4 ± 1.0	29.3 ± 0.6	35.8 ± 2.3	34.4 ± 0.8
After 12 weeks	34.6 ± 1.1	29.6 ± 1.0	35.8 ± 2.5	34.8 ± 1.3

Data are presented as mean ± SEM. ND, normal diet (*n* = 5); NDU, normal diet + uninephrectomy (*n* = 5); HD, high-fat diet (*n* = 8); and HDU, high-fat diet + uninephrectomy (*n* = 8).

**Table 3:** Biochemistry parameters of the four mouse groups.

Average	ND	NDU	HD	HDU
Blood urea nitrogen (mg/dL)	23.7 ± 2.1	34.3 ± 0.6 **	20.0 ± 1.8	43.3 ± 3.4 ^#,$$$^
Creatinine (mg/dL)	0.17 ± 0.01	0.24 ± 0.01 *	0.17 ± 0.01	0.23 ± 0.03 ^$^
Cholesterol (mg/dL)	113.0 ± 2.5	101.2 ± 2.8	158.3 ± 2.4 ***	164.6 ± 6.7 ^###^
LDL cholesterol (mg/dL)	23.2 ± 3.4	12.2 ± 0.6	46.3 ± 4.5 ***	37.7 ± 2.5 ^###^
HDL cholesterol (mg/dL)	75.4 ± 3.1	82.5 ± 2.3	90.0 ± 7.3	121.2 ± 9.3 ^###,$$$^
TG (mg/dL)	140.2 ± 14.5	106.2 ± 7.5	123.7 ± 22.7	140.7 ± 53.7
Glucose (mg/dL)	202.4 ± 17.1	248.1 ± 10.1	199.3 ± 30.7	235.2 ± 30.4
Albumin (g/dL)	2.7 ± 0.2	3.5 ± 0.0 **	2.8 ± 0.2	3.2 ± 0.2
Protein (g/dL)	4.3 ± 0.1	4.8 ± 0.0 *	4.6 ± 0.1	4.6 ± 0.1
ALT (U/L)	46.2 ± 9.7	20.2 ± 0.9 *	35.3 ± 5.0	36.3 ± 8.5
AST (U/L)	137.4 ± 40.1	64.0 ± 6.3	142.0 ± 18.5	132.8 ± 26.7

Data are presented as mean ± SEM. * *p* < 0.05, ** *p* < 0.01, *** *p* < 0.001 versus ND; ^#^ *p* < 0.05, ^###^ *p* < 0.001 versus NDU; ^$^ *p* < 0.05, ^$$$^ *p* < 0.001 versus HD. ND, normal diet (*n* = 5); NDU, normal diet + uninephrectomy (*n* = 5); HD, high-fat diet (*n* = 8); and HDU, high-fat diet + uninephrectomy (*n* = 8).

**Table 4:** Significantly differentially expressed genes based on transcriptome sequencing.

			Log-Fold Change				
Gene Name Abbreviation	Gene Full Name	Pathway	NDU vs. ND	HD vs. ND	HDU vs. ND	HDU vs. HD	HDU vs. NDU
*Rnf20*	Ring finger protein 20	Protein modification and ubiquitination	1.08	−1.02	−1.03	−1.00	−1.11
*Ube2ql1*	Ubiquitin-conjugating enzyme E2Q family-like 1	Ubiquitin-mediated proteolysis	−2.57 *	−1.16	−3.07 **	−2.65 ^##^	−1.2
*NcoR1*	Nuclear receptor corepressor 1	Nuclear receptor transcription pathway	1.35 **	−1.02	−1.03	−1.02	−1.40 ^$$^
*NcoR2*	Nuclear receptor corepressor 2	Nuclear receptor transcription pathway	−1.13	−1.09	−1.06	1.03	1.06
*Pparα*	Peroxisome proliferator-activated receptor alpha	PPAR signaling	1.38 *	2.06 **	1.83 **	−1.13	1.32
*Pparζ*	Peroxisome proliferator-activator receptor delta	PPAR signaling	−1.06	−2.23 **	−1.54 *	1.45	−1.45
*Pparγ*	Peroxisome proliferator-activated receptor gamma	PPAR signaling	−9.45 **	1.28	−2.76	−3.53	3.42
*Angptl4*	Angiopoietin-like 4	PPAR signaling	1.93 *	2.17 **	3.62 **	1.67 ^#^	1.88 ^$^
*Apoa1*	Apolipoprotein A-I	PPAR signaling	−1.33	−3.18 **	2.20 *	7.01 ^##^	2.94 ^$$^
*Apoc3*	Apolipoprotein C-III	PPAR signaling	−1.13	−3.53 **	1.28	4.51 ^##^	1.45
*Hmgcs2*	3-hydroxy-3-methylglutaryl- coenzyme A synthase 2	PPAR signaling	2.95 **	4.19 **	2.92 **	−1.44	−1.01
*Pck1*	Phosphoenolpyruvate carboxykinase 1, cytosolic	PPAR signaling	1.22	2.53 **	3.45 **	1.36	2.84 ^$$^
*Plin4*	perilipin 4	PPAR signaling	−13.5 **	2.83	−2.47	−6.98 ^#^	5.47
*Pltp*	Phospholipid transfer protein	PPAR signaling	−1.67	3.06 *	−2.3	−7.04 ^##^	−1.38
*Cpt1b*	Carnitine palmitoyltransferase 1b	PPAR signaling Fatty acid degradation	−2.62 *	1.82	−1.35	−2.47	1.94
*Cpt1c*	Carnitine palmitoyltransferase 1c	PPAR signaling Fatty acid metabolism	−1.13	1.06	−2.04 **	−2.17 ^##^	−1.80 ^$$^
*Scd1*	Stearoyl-Coenzyme A desaturase 1	PPAR signaling Fatty acid metabolism	−7.04 **	−1.93	−5.43 *	−2.81	1.3
*Scd2*	Stearoyl-Coenzyme A desaturase 2	PPAR signaling Fatty acid metabolism	−1.91	−1.24	−2.70 **	−2.18 ^#^	−1.42
*Acacα*	Acetyl-coenzyme A carboxylase alpha	Fatty acid metabolism	−9.70 **	−2.13	−5.82 *	−2.74	1.67
*Fasn*	Fatty acid synthase	Fatty acid metabolism	−14.92 **	−1.86	−6.57 *	−3.54	2.27
*Hacd4*	3-hydroxyacyl-CoA dehydratase 4	Fatty acid metabolism	−4.48 *	1.1	−4.67 **	−5.16 ^##^	−1.04
*Acaa2*	Acetyl-coenzyme A acyltransferase 2	Fatty acid degradation	−1.04	1.42	−1.54 *	−2.19 ^##^	−1.48
*Cyp2u1*	Cytochrome P450, family 2, subfamily u, polypeptide 1	Fatty acid degradation	−1.08	1.57 *	−1.58 *	−2.48 ^##^	−1.46
*Atp1α2*	ATPase, Na^+^/K^+^ transporting, alpha 2 polypeptide	PT bicarbonate reclamation	−15.66 **	1.62	−3.61	−5.83 ^#^	4.34
*Atp1α3*	ATPase, Na^+^/K^+^ transporting, alpha 3 polypeptide	PT bicarbonate reclamation	−11.15 **	−1.42	−7.22 *	−5.09	1.55
*Atp1β2*	ATPase, Na^+^/K^+^ transporting, beta 2 polypeptide	PT bicarbonate reclamation	−1.43 *	1.02	−2.51 **	−2.55 ^##^	−1.75 ^$$^
*Cebpβ*	CCAAT/enhancer-binding protein (C/EBP), beta	TNF signaling	−1.46	−1.2	2.22 **	2.67 ^##^	3.23 ^$$^
*Adcy3*	Adenylate cyclase 3	Vasopressin-regulated water reabsorption	−3.35 *	1.48	−3.08 *	−4.57 ^##^	1.09
*Aqp2*	Aquaporin 2	Vasopressin-regulated water reabsorption	−2.37 **	−1.2	1.1	1.32	2.61 ^$$^
*Aqp4*	Aquaporin 4	Vasopressin-regulated water reabsorption	−3.45 *	1.14	−1.33	−1.51	2.6
*Arhgdiγ*	Rho-GDP dissociation inhibitor gamma	Vasopressin-regulated water reabsorption	−4.20 **	1.5	−1.08	−1.62	3.88 ^$$^
*Avpr2*	Arginine vasopressin receptor 2	Vasopressin-regulated water reabsorption	−1.52	−1.08	−2.55 **	−2.37 ^##^	−1.68 ^$^
*Crebbp*	CREB-binding protein	TGF-beta signaling, cell cycle, JAK-STAT signaling	−1.04	−1.12	−1.08	1.04	−1.04
*Abca1*	ATP-binding cassette, subfamily A, member 1	ABC transporters, Fat digestion and absorption, cholesterol metabolism	1.03	1.38	−1.39	−1.92 ^##^	−1.43
*Acox1*	Acyl-coenzyme A oxidase 1	Fatty acid degradation unsaturated fatty acid biosynthesis	1.35 *	1.11	1.46 **	1.32 ^#^	1.08
*Ar*	Androgen receptor	Androgen receptor signaling	1.36 *	1.06	1.59 **	1.49 ^#^	1.16

* *p* < 0.05, ** *p* < 0.01 versus ND; ^#^ *p* < 0.05, ^##^ *p* < 0.01 versus HD; ^$^ *p* < 0.05, ^$$^ *p* < 0.01 versus NDU. *n* = 3 for each group.

## Data Availability

Data are contained within the article.

## References

[1] Escasany E., Izquierdo-Lahuerta A., Medina-Gómez G., del Moral A.M., Aguilera García C.M. (2018). Chapter 7—Kidney Damage in Obese Subjects: Oxidative Stress and Inflammation. Obesity.

[2] Gai Z., Wang T., Visentin M., Kullak-Ublick G.A., Fu X., Wang Z. (2019). Lipid Accumulation and Chronic Kidney Disease. Nutrients.

[3] Stasi A., Cosola C., Caggiano G., Cimmarusti M.T., Palieri R., Acquaviva P.M., Rana G., Gesualdo L. (2022). Obesity-Related Chronic Kidney Disease: Principal Mechanisms and New Approaches in Nutritional Management. Front. Nutr..

[4] Herrington W.G., Smith M., Bankhead C., Matsushita K., Stevens S., Holt T., Hobbs F.D.R., Coresh J., Woodward M. (2017). Body-mass index and risk of advanced chronic kidney disease: Prospective analyses from a primary care cohort of 1.4 million adults in England. PLoS ONE.

[5] Wang X., Xing C., Li G., Dai X., Gao X., Zhuang Y., Cao H., Hu G., Guo X., Yang F. (2023). The key role of proteostasis at mitochondria-associated endoplasmic reticulum membrane in vanadium-induced nephrotoxicity using a proteomic strategy. Sci. Total Environ..

[6] Gilbert R.E. (2017). Proximal Tubulopathy: Prime Mover and Key Therapeutic Target in Diabetic Kidney Disease. Diabetes.

[7] Edwards A., Palm F., Layton A.T. (2020). A model of mitochondrial O2 consumption and ATP generation in rat proximal tubule cells. Am. J. Physiol. Ren. Physiol..

[8] Forst A.L., Reichold M., Kleta R., Warth R. (2021). Distinct Mitochondrial Pathologies Caused by Mutations of the Proximal Tubular Enzymes EHHADH and GATM. Front. Physiol..

[9] Chen L., Higgins P.J., Zhang W. (2017). Development and Diseases of the Collecting Duct System. Results Probl. Cell Differ..

[10] Shiloh Y., Shema E., Moyal L., Oren M. (2011). RNF20-RNF40: A ubiquitin-driven link between gene expression and the DNA damage response. FEBS Lett..

[11] Zhang M., Su L., Wang W., Li C., Liang Q., Ji F., Jiao J. (2022). Endothelial cells regulated by RNF20 orchestrate the proliferation and differentiation of neural precursor cells during embryonic development. Cell Rep..

[12] Zhao Y., Pan J., Cao C., Liang X., Yang S., Liu L., Tao C., Zhao J., Wang Y. (2021). RNF20 affects porcine adipocyte differentiation via regulation of mitotic clonal expansion. Cell Prolif..

[13] Lee J.H., Lee G.Y., Jang H., Choe S.S., Koo S.-H., Kim J.B. (2014). Ring finger protein20 regulates hepatic lipid metabolism through protein kinase A-dependent sterol regulatory element binding protein1c degradation. Hepatology.

[14] Jeon Y.G., Lee J.H., Ji Y., Sohn J.H., Lee D., Kim D.W., Yoon S.G., Shin K.C., Park J., Seong J.K. (2020). RNF20 Functions as a Transcriptional Coactivator for PPARγ by Promoting NCoR1 Degradation in Adipocytes. Diabetes.

[15] Zhang X.-Y., Wang B., Guan Y.-F. (2016). Nuclear Receptor Regulation of Aquaporin-2 in the Kidney. Int. J. Mol. Sci..

[16] Corrales P., Izquierdo-Lahuerta A., Medina-Gómez G. (2018). Maintenance of Kidney Metabolic Homeostasis by PPAR Gamma. Int. J. Mol. Sci..

[17] Kong Y., Feng W., Zhao X., Zhang P., Li S., Li Z., Lin Y., Liang B., Li C., Wang W. (2020). Statins ameliorate cholesterol-induced inflammation and improve AQP2 expression by inhibiting NLRP3 activation in the kidney. Theranostics.

[18] Mount P., Davies M., Choy S.-W., Cook N., Power D. (2015). Obesity-Related Chronic Kidney Disease—The Role of Lipid Metabolism. Metabolites.

[19] Bulbul M.C., Dagel T., Afsar B., Ulusu N.N., Kuwabara M., Covic A., Kanbay M. (2018). Disorders of Lipid Metabolism in Chronic Kidney Disease. Blood Purif..

[20] Sethi G., Shanmugam M.K., Arfuso F., Kumar A.P. (2018). Role of RNF20 in cancer development and progression—A comprehensive review. Biosci. Rep..

[21] Liang X., Tao C., Pan J., Zhang L., Liu L., Zhao Y., Fan Y., Cao C., Liu J., Zhang J. (2021). Rnf20 deficiency in adipocyte impairs adipose tissue development and thermogenesis. Protein Cell.

[22] Lee J.H., Jeon Y.G., Lee K.H., Lee H.W., Park J., Jang H., Kang M., Lee H.S., Cho H.J., Nam D.H. (2017). RNF20 Suppresses Tumorigenesis by Inhibiting the SREBP1c-PTTG1 Axis in Kidney Cancer. Mol. Cell Biol..

[23] Geiger M.A., Guillaumon A.T., Paneni F., Matter C.M., Stein S. (2020). Role of the Nuclear Receptor Corepressor 1 (NCOR1) in Atherosclerosis and Associated Immunometabolic Diseases. Front. Immunol..

[24] Luan Z.L., Zhang C., Ming W.H., Huang Y.Z., Guan Y.F., Zhang X.Y. (2022). Nuclear receptors in renal health and disease. EBioMedicine.

[25] Gao J., Gu Z. (2022). The Role of Peroxisome Proliferator-Activated Receptors in Kidney Diseases. Front. Pharmacol..

[26] Sharma V., Patial V. (2022). Peroxisome proliferator-activated receptor gamma and its natural agonists in the treatment of kidney diseases. Front. Pharmacol..

[27] Kwon T.H., Frøkiær J., Nielsen S. (2013). Regulation of aquaporin-2 in the kidney: A molecular mechanism of body-water homeostasis. Kidney Res. Clin. Pract..

